# Waterpipe Smoke Condensate Impairs p53 Nuclear Localization in Association with HIF-1α Stabilization

**DOI:** 10.3390/ijms27198927

**Published:** 2026-10-08

**Authors:** Husam Nawafleh, Jerome Thiery, Yehya Elsayed, Abderemane Abdou, Salem Chouaib, Rania F. Zaarour

**Affiliations:** 1 Thumbay Research Institute for Precision Medicine, Gulf Medical University, Al Jurf 1, Ajman P.O. Box 4184, United Arab Emirates; husam@gmu.ac.ae (H.N.); salem.chouaib@gmu.ac.ae (S.C.); 2 College of Medicine, Gulf Medical University, Al Jurf 1, Ajman P.O. Box 4184, United Arab Emirates; 3 INSERM UMR 1356, Next-Generation Immuno-Oncology Research and Therapy in Pediatric and Adult Cancer, Gustave Roussy, Faculty of Medicine, Université Paris-Saclay, 114 Rue Édouard-Vaillant, 94805 Villejuif, France; jerome.thiery@gustaveroussy.fr (J.T.); abderamane.abdou@gustaveroussy.fr (A.A.); 4 Department of Chemistry, Wayne State University, 5101 Cass Avenue, Detroit, MI 48202, USA; hy5488@wayne.edu

**Keywords:** smoking, waterpipe, HIF-1α, p53, lung cancer, hypoxia

## Abstract

Waterpipe (shisha or hookah) smoking is recognized as a risk factor for lung cancer; however, its interference with hypoxia stress and tumor suppressor pathways in non-small cell lung cancer (NSCLC) is poorly defined. In this study, NSCLC cell lines (A549, H460, H226 with endogenous wild-type p53, and H1299 transiently expressing wild-type or p53 mutants) were exposed to waterpipe smoke condensate (WPSC) under normoxic and/or hypoxic conditions. WPSC induced HIF-1α stabilization under normoxic conditions, accompanied by predominantly cytoplasmic localization and without corresponding induction of the canonical HIF-1 target genes examined. In parallel, WPSC transiently reduced nuclear p53 localization without altering total p53 protein levels. In A549 cells, WPSC pretreatment attenuated irradiation-induced expression of the p53 target genes cyclin-dependent kinase inhibitor 1A (*CDKN1A*) and BTG anti-proliferation factor 2 (*BTG2*). Pharmacological inhibition of HIF-1α partially restored nuclear p53 localization, supporting the involvement of HIF-1α in this response. Co-immunoprecipitation demonstrated an association between HIF-1α and p53 following WPSC exposure. Collectively, these findings show that WPSC-induced HIF-1α stabilization under normoxic conditions is associated with transient alterations in p53 localization and transcriptional response, supporting an involvement of HIF-1α in this process.

## 1. Introduction

Lung cancer is the leading cause of cancer-related mortality worldwide, and non-small cell lung cancer (NSCLC) accounts for approximately 85% of all cases [1]. While cigarette smoking is the best-established risk factor for lung cancer [2], increasing evidence suggests that alternative forms of tobacco consumption, including the globally popular waterpipe (shisha or hookah), are also implicated in lung cancer development. Waterpipe smoke contains high levels of toxicants, including carbon monoxide, polycyclic aromatic hydrocarbons, and reactive oxygen species, all of which contribute to carcinogenesis and development of lung cancer [3]. The molecular mechanisms by which waterpipe smoke influences tumor biology are still poorly understood and warrant further investigation.

A key feature of the tumor microenvironment is hypoxia, a condition of reduced oxygen availability that drives cancer progression, metabolic reprogramming, and resistance to therapy [4]. Cellular adaptation to hypoxia is primarily mediated by hypoxia-inducible factor 1-alpha (HIF-1α), a transcription factor whose stability is tightly regulated by oxygen levels [5]. Under normoxic conditions, HIF-1α is rapidly degraded, whereas hypoxia stabilizes the protein, allowing nuclear translocation and transcriptional activation of genes involved in angiogenesis, glycolysis, and cell survival [6]. Interestingly, several environmental stress factors, including cigarette smoke exposure, have been reported to induce pseudohypoxia, a state in which HIF-1α is stabilized independently of low oxygen levels [7,8]. Whether waterpipe smoke exerts similar effects in lung cancer cells remains unexplored.

The tumor suppressor p53 plays a central role in maintaining genomic integrity by detecting and responding to DNA damage, subsequently regulating cell cycle arrest, DNA repair and apoptosis. The activity of wild-type p53 is tightly regulated at the level of protein stability and subcellular localization, as its role as a transcriptional regulator requires nuclear accumulation [9]. Importantly, crosstalk between HIF-1α and p53 has been documented, with evidence suggesting that HIF-1α can modulate p53 stability, nuclear localization and transcriptional activity in a context-dependent manner [10,11], thereby influencing cell fate decisions [11].

Our previous study demonstrated that long-term waterpipe smoke condensate (WPSC) exposure modulates cellular stress and survival-related signaling pathways, highlighting the capacity of waterpipe smoke to induce sustained biological responses in lung cancer cells under chronic exposure conditions [12].

Despite these insights, the interplay between waterpipe smoke exposure, hypoxia signaling, and p53 function in NSCLC has not been clearly defined. In particular, it remains unknown whether waterpipe smoke can induce HIF-1α stabilization under normoxic conditions, how this impacts HIF-1α activity, and whether p53 tumor suppressor pathway is affected.

In this study, we examined whether WPSC induces HIF-1α stabilization under normoxic conditions and whether this response is associated with changes in p53 localization and activity in NSCLC (A549, H460, and H226) cells. These cells were selected to represent distinct NSCLC histological backgrounds while retaining endogenous wild-type p53.

## 2. Results

### 2.1. WPSC Induces Stabilization of HIF-1α Without Corresponding Transcriptional Activation

**Figure 1:**
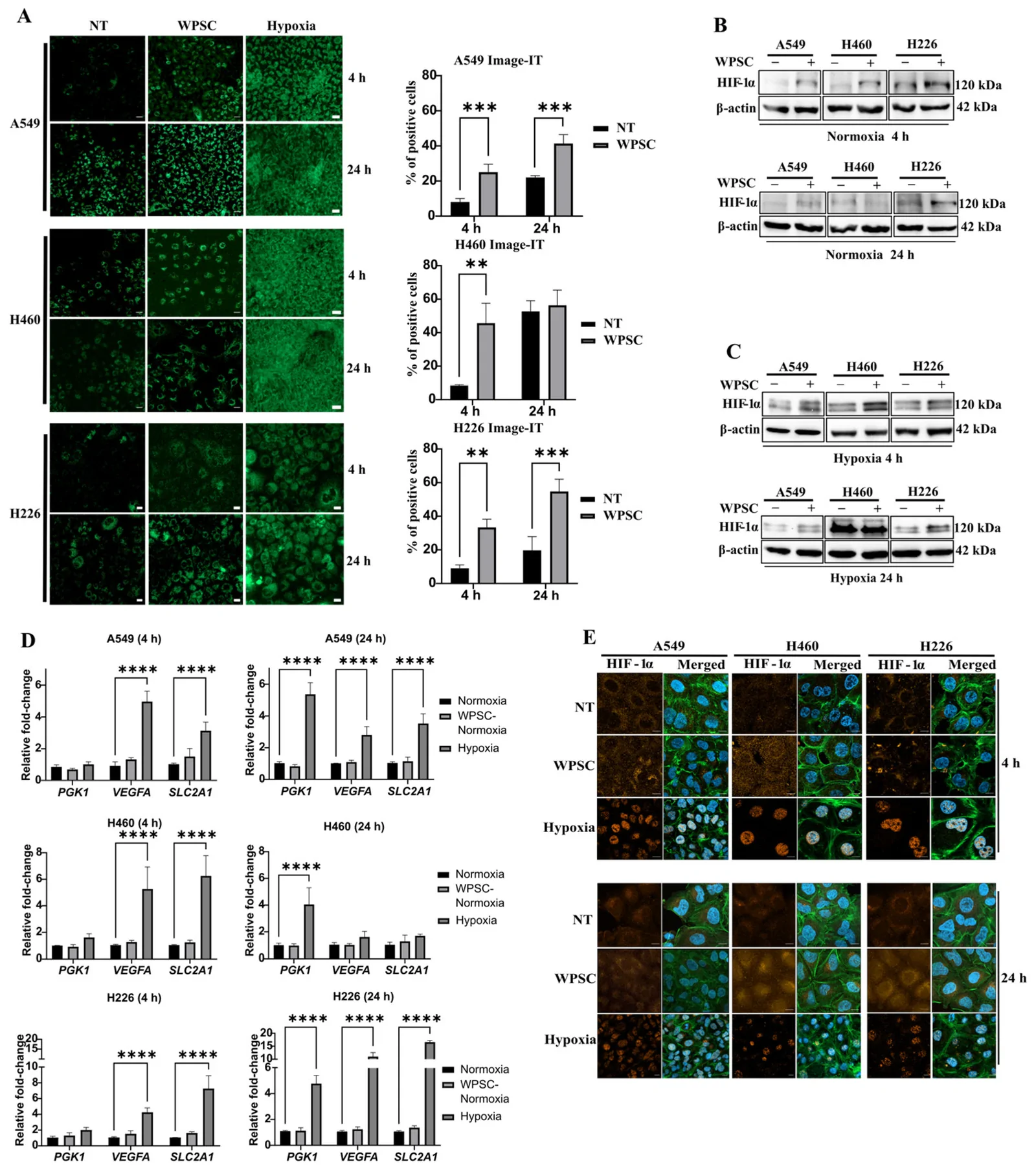
WPSC induces HIF-1α stabilization. (**A**) Representative confocal images from the 4 h and 24 h time points are displayed (left panel). Scale bar, 20 μm. Quantification of green-positive cells is shown (right panel). Representative images show individual microscopic fields, whereas quantification was performed across multiple fields from three independent experiments (>200 cells per condition). (**B**,**C**) Western blot analysis of HIF-1α under the indicated conditions. (**D**) RT-qPCR analysis of *VEGFA*, *PGK1*, and *SLC2A1*. (**E**) Immunofluorescence analysis of HIF-1α subcellular localization under the indicated conditions (HIF-1α in orange, actin in green and nucleus in blue). Scale bar, 10 μm. Quantitative data are presented as the mean ± SD from at least three independent experiments. For panels (**A**,**D**), statistical analysis was performed using two-way ANOVA followed by the Benjamini–Hochberg false discovery rate (FDR) procedure for multiple comparisons. In panel (**A**), NT and WPSC-treated cells were compared at each time point. In panel (**D**), WPSC-treated and hypoxic cells were compared with the corresponding normoxic control within each gene at the indicated time points. NT: non-treated, WPSC: waterpipe smoke condensate. Statistical significance was determined at an FDR of 5% (q ≤ 0.05). ** q ≤ 0.01, *** q ≤ 0.001 and **** q ≤ 0.0001.

Because cigarette smoke has been reported to induce hypoxic responses in lung tissues [7], we examined whether WPSC could induce hypoxia-associated cellular responses in NSCLC cells. For this purpose, lung cancer cell lines A549, H460, and H226 were exposed to 0.1% WPSC for 4 h or 24 h and stained with the Image-iT™ Hypoxia Reagent Green dye to assess the induction of hypoxia-like cellular responses. The 0.1% WPSC concentration was selected based on dose–response MTT experiments in A549, H460, and H226 cells, which showed no reduction in MTT signal at 0.1% over 24–72 h, whereas reductions were observed at higher concentrations. This concentration was therefore used to investigate molecular responses while minimizing potential effects associated with reduced cell viability. Following 4 h WPSC treatment, all three cell lines displayed a significant increase in Image-iT™-positive staining compared to untreated controls. The percentage of positive cells increased from 10% to 30% in A549 and H226 cells and from 18% to 45% in H460 cells. Following 24 h treatment, A549 and H226 cells maintained a significant increase in Image-iT™-positive staining (45% and 40%, respectively), whereas H460 cells showed no significant increase. As a positive control, exposure to hypoxia resulted in near-complete staining (95–100%) in all cell lines (Figure 1A).

We next examined whether the Image-iT™ signal was accompanied by HIF-1α stabilization. HIF-1α stabilization was observed in all three cell lines following 4 h WPSC treatment under normoxic conditions. Following 24 h of WPSC treatment, HIF-1α protein levels were maintained in A549 and H226 cells; however, H460 cells no longer showed increased protein levels compared to non-treated cells (Figure 1B). Because WPSC induced HIF-1α stabilization under normoxic conditions, we examined whether exposure to hypoxia (1% O_2_), along with WPSC, could further increase HIF-1α levels beyond the level induced by hypoxia alone. In line with observed HIF-1α stabilization, cells exposed to hypoxia in combination with WPSC showed higher HIF-1α levels compared with hypoxia alone at 4 h and in A549 and H226 at 24 h (Figure 1C).

Finally, we assessed the transcriptional activity of HIF-1α by measuring the expression of selected canonical HIF-1α target genes, vascular endothelial growth factor A (*VEGFA*), phosphoglycerate kinase 1 (*PGK1*), and solute carrier family 2 member 1 (*SLC2A1*), following WPSC treatment. Surprisingly, RT-qPCR analysis did not reveal an increased expression of these genes (Figure 1D). Accordingly, we investigated whether HIF-1α was able to translocate to the nucleus. Immunofluorescence analysis showed that, in contrast to hypoxia, which promoted clear nuclear HIF-1α accumulation, WPSC-stabilized HIF-1α remained predominantly cytoplasmic (Figure 1E).

Together, our results indicate that WPSC stabilizes HIF-1α under normoxic conditions, with predominantly cytoplasmic localization and no corresponding induction of the canonical HIF-1 target genes examined.

### 2.2. WPSC Transiently Reduces Nuclear p53 Localization in NSCLC Cells and Attenuates Irradiation-Induced p53 Target Gene Expression in A549 Cells

**Figure 2:**
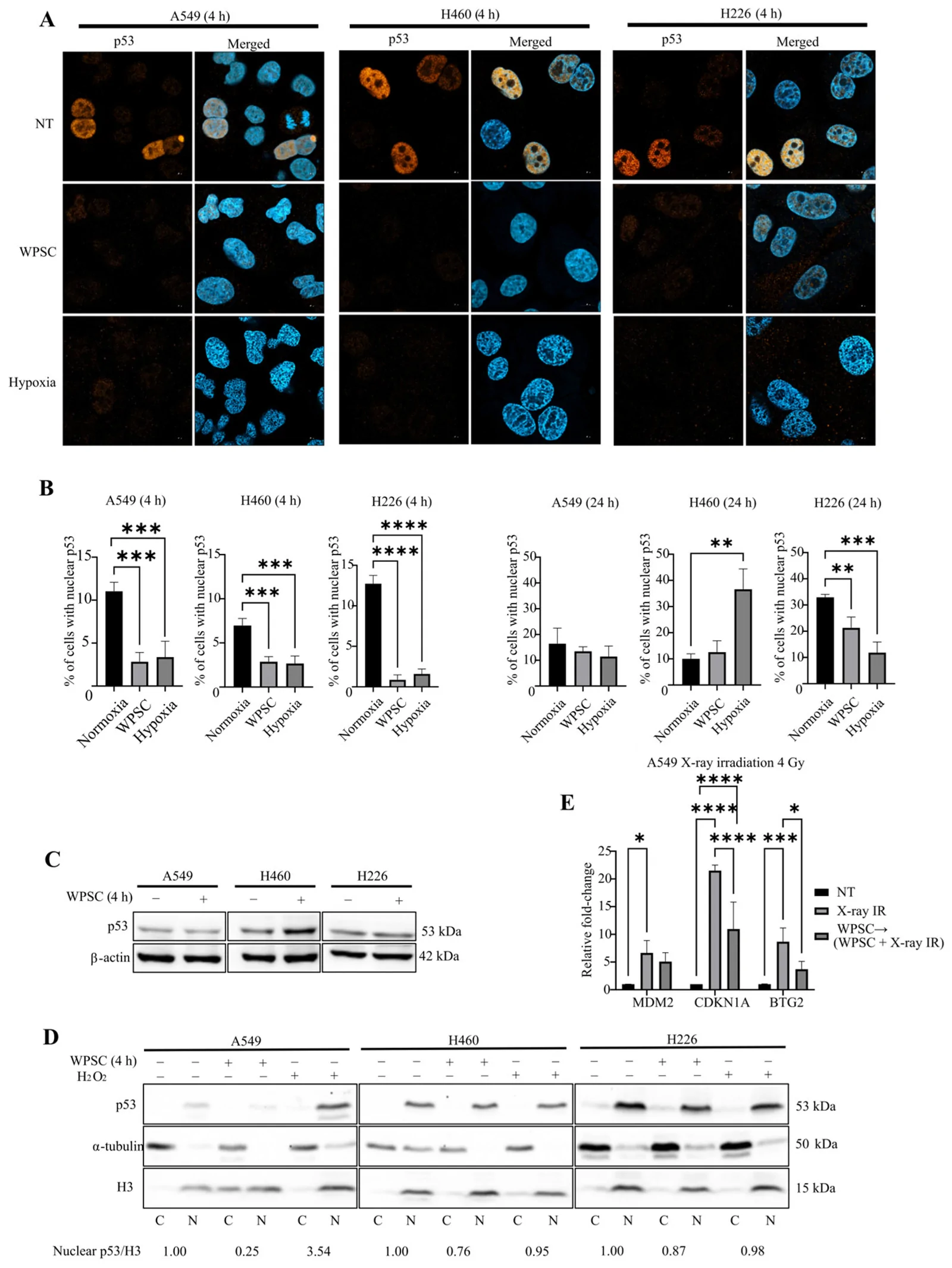
WPSC transiently reduces nuclear p53 localization in NSCLC cells and attenuates irradiation-induced p53 target gene expression in A549 cells. (**A**) Representative immunofluorescence confocal images of p53 in A549, H460, and H226 cells under the indicated conditions. Scale bar, 10 μm. (p53 in orange and nucleus in blue). (**B**) Quantification of nuclear p53 in all three cell lines under the indicated conditions and time points. Over 100 cells were analyzed from at least three independent experiments. (**C**) Western blot of total p53 protein under the indicated conditions. (**D**) Cytoplasmic–nuclear fractionation analysis of p53. Histone H3 was utilized as a nuclear marker, and α-tubulin was utilized as a cytoplasmic marker. H_2_O_2_ served as a positive control. (**E**) RT-qPCR analysis of the p53 target genes (*MDM2*, *CDKN1A*, and *BTG2*) following irradiation with or without WPSC treatment. Values indicate nuclear p53 normalized to Histone H3 and expressed relative to the corresponding non-treated (NT) control. Quantitative data are presented as the mean ± SD from at least three independent experiments. For panel (**B**), statistical analysis was performed using one-way ANOVA followed by the Benjamini–Hochberg false discovery rate (FDR) procedure for multiple comparisons, comparing WPSC-treated and hypoxic cells with the corresponding normoxic control. Statistical significance was determined at an FDR of 5% (q ≤ 0.05). For panel (**E**), statistical analysis was performed using two-way ANOVA followed by Tukey’s multiple-comparisons test, comparing treatment conditions within each gene. Adjusted *p* ≤ 0.05 was considered statistically significant. ** q ≤ 0.01 and *** q ≤ 0.001 **** q ≤ 0.0001 for panel (**B**); * *p* ≤ 0.05, *** *p* ≤ 0.001 and **** *p* ≤ 0.0001 for panel (**E**).

To investigate the effects of HIF-1α cytoplasmic stabilization on downstream signaling, we investigated whether WPSC exposure alters the nuclear localization and transcriptional activity of wild-type (WT) p53, given the documented crosstalk between HIF-1α and p53 signaling. Immunofluorescence analysis demonstrated that 4 h of WPSC exposure significantly decreased the percentage of cells with nuclear WT p53 compared with non-treated cells: A549 (from 10% to 2%), H460 (from 9% to 3%), and H226 (from 13% to 3%). This is consistent with the effect of hypoxia on p53 nuclear localization in these cells. This reduction was transient, as nuclear p53 localization returned to levels comparable to untreated cells following 24 h of WPSC exposure (Figure 2A,B).

To determine whether the reduction in nuclear p53 was associated with changes in its overall protein abundance, we examined the effects of WPSC on total p53. Results showed that there was no change in total p53 following 4 h WPSC treatment (Figure 2C), suggesting that the decrease in nuclear p53 occurred independently of changes in protein abundance. In addition, nuclear-cytoplasmic fractionation confirmed a marked reduction in nuclear p53 following 4 h of WPSC exposure, with H_2_O_2_ serving as a positive control for p53 nuclear accumulation (Figure 2D).

We next assessed whether the observed reduction in nuclear p53 was associated with changes in transcriptional activity. Expression of canonical p53 target genes MDM2 proto-oncogene (*MDM2*), cyclin-dependent kinase inhibitor 1A (*CDKN1A*), and BTG anti-proliferation factor 2 (*BTG2*) following 4 h of WPSC treatment was not significantly reduced compared with non-treated cells in any of the examined cell lines (Figure S1), likely reflecting the low proportion of cells with nuclear p53 (~10%). To increase the nuclear p53 fraction, cells were exposed to 4 Gy irradiation, a moderate dose previously shown to activate p53-related responses in NSCLC [13]. In A549 cells, irradiation significantly increased the expression of *MDM2*, *CDKN1A*, and *BTG2*, whereas WPSC pretreatment significantly decreased irradiation-induced expression of the p53 target genes *CDKN1A* and *BTG2* compared with irradiation alone (Figure 2E). In contrast, in H460 and H226, we observed minimal or no upregulation of p53 downstream genes 5 h after (4 Gy) irradiation (Figure S2). This is not surprising given that H460 is known to exhibit delayed p53 activation after moderate irradiation, with robust induction only at later time points (24 h) [14]. In H226, early induction of p53 target genes has not been characterized; however, apoptosis remains minimal at 48 h following irradiation [15].

Together, our results demonstrated that WPSC transiently reduces nuclear p53 localization across the examined NSCLC models and attenuates irradiation-induced p53 target gene expression in A549 cells.

### 2.3. WPSC Differentially Affects the Nuclear Localization of p53 Mutants

**Figure 3:**
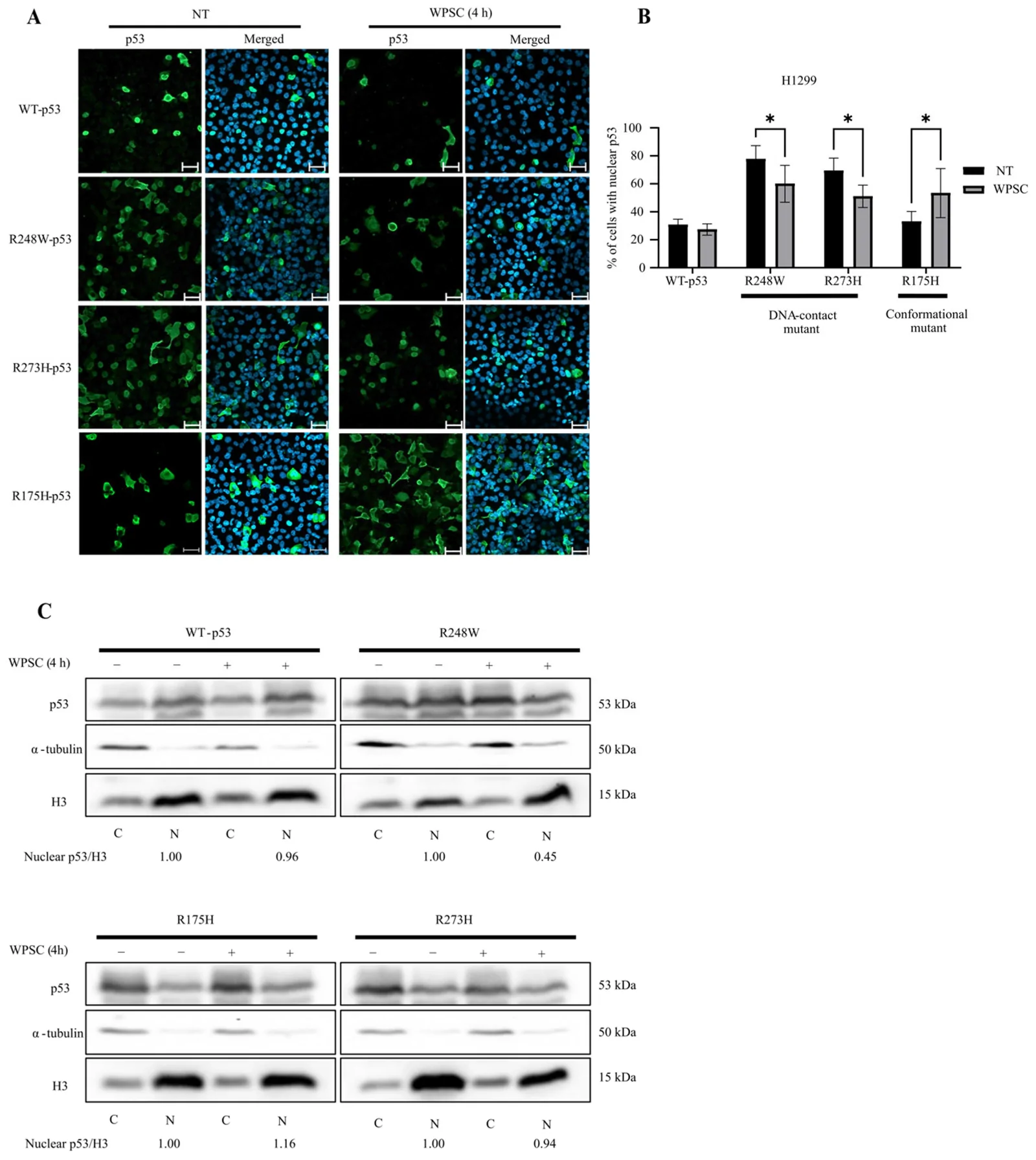
WPSC modulates nuclear localization of mutant p53 in H1299 transfected cells. (**A**) Representative immunofluorescence confocal images of H1299 cells expressing wild-type or mutant p53 (R248W, R273H, and R175H) under the indicated conditions. (p53 in green and nucleus in blue). Scale bar, 20 μm. (**B**) Quantification of nuclear p53. (**C**) Subcellular p53 localization assessed by cytoplasmic–nuclear fractionation for WT and all mutant variants (R248W, R273H, and R175H). Histone H3 was utilized as a nuclear marker, and α-tubulin was utilized as a cytoplasmic marker. Values indicate nuclear p53 normalized to Histone H3 and expressed relative to the corresponding non-treated (NT) control. All quantitative data are mean ± SD from at least three independent experiments. Statistical analysis was performed using two-way ANOVA followed by uncorrected Fisher’s least significant difference (LSD) test, comparing NT and WPSC-treated cells within each p53 variant. * *p* ≤ 0.05.

To examine whether the effect of WPSC on p53 subcellular localization varies among p53 mutants, p53-null H1299 cells were transfected with WT p53, structural mutant R175H, or DNA-contact mutants R248W and R273H [16]. Immunofluorescence analysis showed that the effect of WPSC on nuclear p53 localization differed according to the p53 variant. WPSC significantly reduced nuclear localization of DNA-contact mutants R248W and R273H. In contrast, nuclear localization of conformational mutant R175H increased following WPSC treatment (Figure 3A,B). Consistent with these observations, nuclear-cytoplasmic fractionation further demonstrated differential effects of WPSC among the p53 variants, with the most pronounced reduction in nuclear p53 observed for R248W (Figure 3C).

Together, these results demonstrated that the effect of WPSC on p53 nuclear localization varies depending on the p53 mutants.

### 2.4. PX-478 Partially Restores Nuclear p53 Localization Following WPSC Exposure

**Figure 4:**
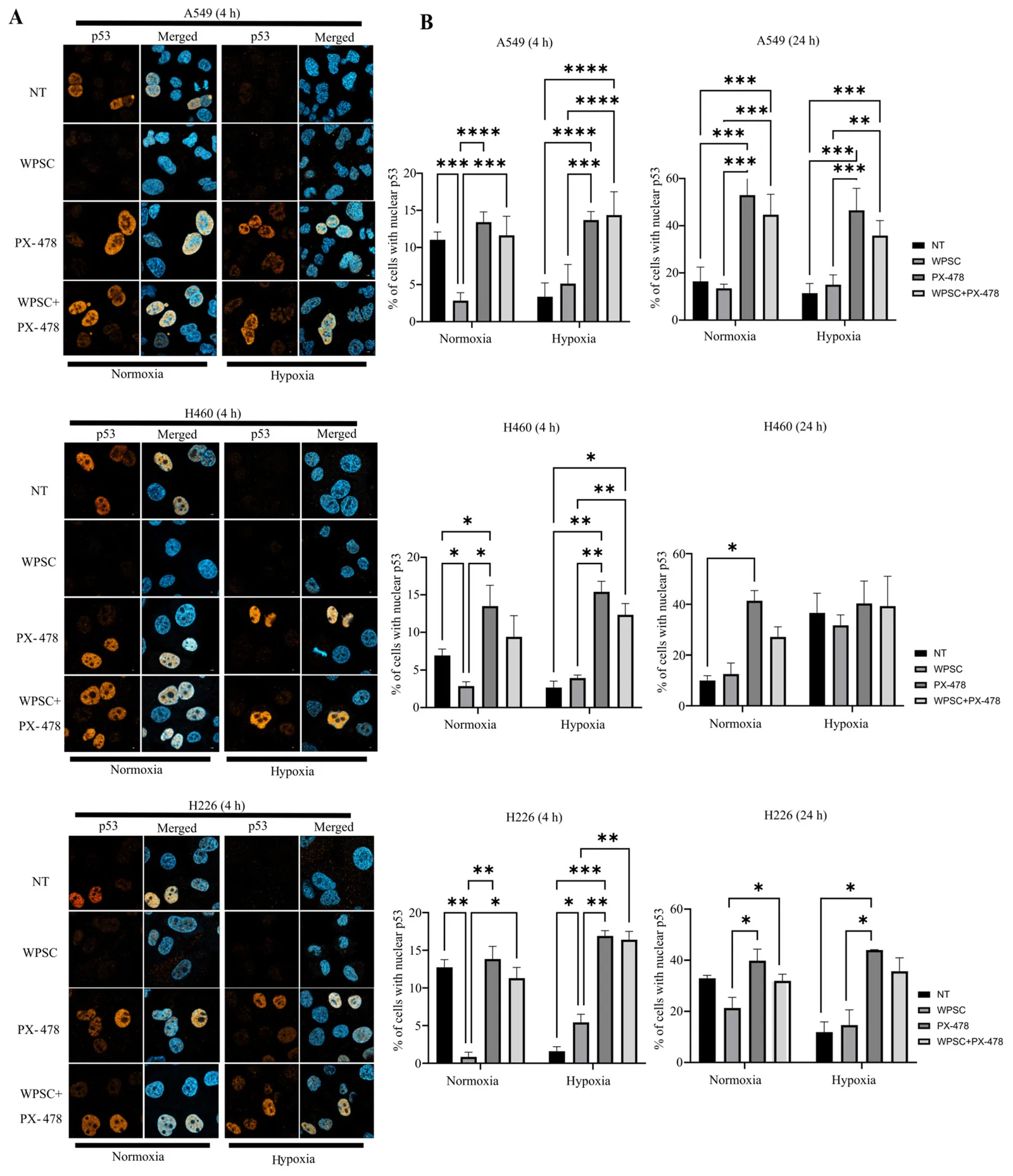
PX-478 alters WPSC-induced changes in nuclear p53 localization. Nuclear p53 localization was assessed by confocal immunofluorescence under the indicated conditions. (**A**) Representative images showing nuclear p53 at 4 h. Scale bar, 10 μm. (p53 in orange and nucleus in blue). (**B**) Quantification of the percentage of cells with nuclear p53. More than 100 cells were analyzed from at least three independent experiments. Data are presented as the mean ± SD from at least three independent experiments. Statistical analysis was performed using two-way ANOVA followed by the Benjamini–Hochberg false discovery rate (FDR) procedure for multiple comparisons within each oxygen condition. Statistical significance was determined at an FDR of 5% (q ≤ 0.05). * q ≤ 0.05, ** q ≤ 0.01, *** q ≤ 0.001 and **** q ≤ 0.0001.

Because WPSC stabilizes HIF-1α and reduces nuclear p53, we next examined whether pharmacological inhibition of HIF-1α with PX-478 altered the WPSC-associated change in p53 localization. A549, H460, and H226 cells were treated with PX-478, a selective HIF-1α inhibitor, alone or in combination with WPSC. PX-478 treatment showed a clear reduction in HIF-1α protein in both normoxic and hypoxic conditions in all three cell lines (Figure S3). Under normoxic conditions, PX-478 treatment restored nuclear p53 in all three cell lines at both 4 h and 24 h, effectively reversing the WPSC-induced reduction. Under hypoxic conditions, PX-478 similarly restored nuclear p53 at 4 h in all three cell lines; however, at 24 h, the effect was observed only in A549 and H226, with no significant change in H460 (Figure 4A,B).

### 2.5. HIF-1α Co-Immunoprecipitates with p53 Following 4 h WPSC Exposure

**Figure 5:**
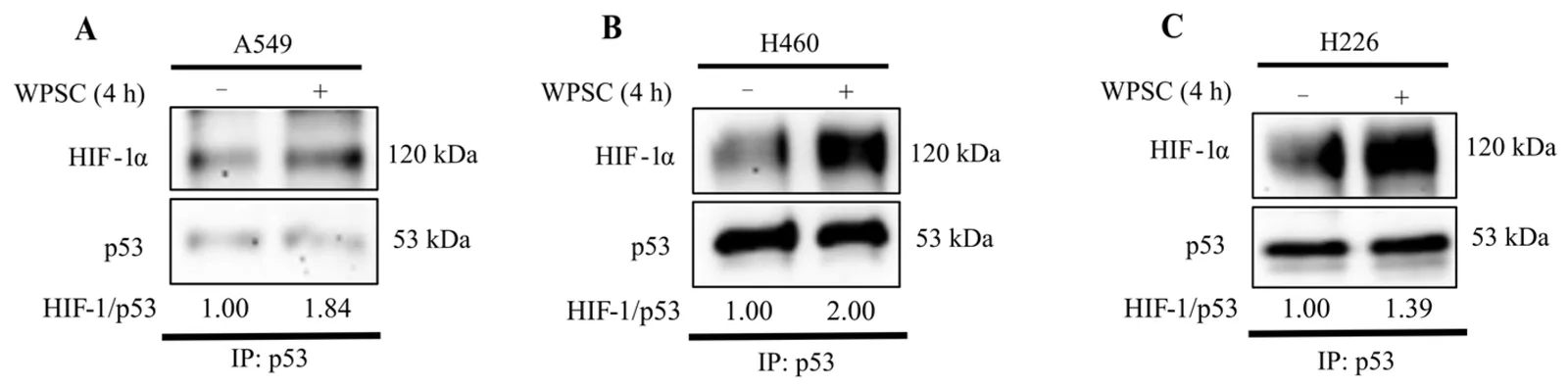
Co-immunoprecipitation analysis of HIF-1α and p53 in (**A**) A549, (**B**) H460, and (**C**) H226 cells under the indicated conditions. Lanes represent non-treated (NT) and WPSC-treated cells. HIF-1α was detected in the p53-immunoprecipitated fraction. Values indicate HIF-1α normalized to p53 and expressed relative to the corresponding non-treated (NT) control.

To investigate whether HIF-1α co-immunoprecipitates with p53 following WPSC exposure, co-immunoprecipitation was performed using whole-cell lysates collected after 4 h of WPSC treatment. HIF-1α was detected in the p53-immunoprecipitated fraction in non-treated cells, with an apparent increase in the HIF-1α signal following WPSC treatment (Figure 5).

## 3. Discussion

In this study, we demonstrated that waterpipe smoke condensate (WPSC) induced a stabilization of HIF-1α under normoxic conditions in NSCLC cells. Consistent with our results, cigarette and waterpipe smoke exposure have been reported to induce systemic hypoxia in humans and in animal models [17,18] and to elicit in vitro responses associated with HIF-1α stabilization [7,19]. Notably, HIF-1α stabilization following WPSC exposure was accompanied by impaired nuclear accumulation and a lack of transcriptional activation. The mechanism underlying WPSC-induced HIF-1α stabilization was not examined in the present study. Previous reports suggest that cigarette smoke can promote HIF-1α accumulation through ROS-dependent signaling and modulation of the PHD/HIF pathway, while waterpipe smoke has also been associated with oxidative stress and mitochondrial alterations [7,20,21]. Whether similar mechanisms contribute to the HIF-1α accumulation observed here remains to be determined. HIF-1α can, under specific conditions, be retained in the cytoplasm; interferon regulatory factor 3 (IRF-3), for example, was recently shown to promote its cytoplasmic retention under hypoxia [22]. Whether IRF-3 or related mechanisms contribute to the WPSC response remains to be investigated.

Extensive crosstalk exists between HIF-1α and p53, whereby HIF-1α modulates p53 activity through direct and indirect interactions and thereby influences p53-dependent transcriptional and apoptotic responses [10]. The activity of p53, a central tumor suppressor, is strongly dependent on its subcellular localization: its best-characterized function is nuclear, where it transcriptionally controls genes governing cell-cycle arrest, DNA repair, senescence, and apoptosis, but it can also act in the cytoplasm and mitochondria through transcription-independent functions [23,24]. Mechanisms that prevent nuclear accumulation of otherwise functional p53 may therefore substantially impair its tumor-suppressive activity.

Short-term WPSC exposure markedly reduced nuclear p53 across multiple NSCLC cell lines without lowering total p53 protein, suggesting that WPSC affects p53 subcellular distribution rather than its overall expression or stability. Tobacco smoke contains stressors that induce oxidative and likely endoplasmic reticulum (ER) stress, and ER stress can promote cytoplasmic localization and destabilization of p53 [25]. Because total p53 was unchanged, however, enhanced degradation is unlikely to explain the phenotype. Instead, our data point to altered intracellular retention or trafficking of p53—an established route, as when cytoplasmic anchoring proteins bind p53 and block its nuclear entry [26]. Consistent with this, WPSC pretreatment attenuated irradiation-induced expression of p53 target genes *CDKN1A* and *BTG2* in A549 cells. However, because H460 and H226 showed little or no induction of the selected p53 targets following irradiation, a comparable transcriptional effect of WPSC could not be established in these two cell lines.

Our results support an association between WPSC-induced HIF-1α stabilization and altered p53 localization. Co-immunoprecipitation showed an association between HIF-1α and p53 following WPSC exposure, while pharmacological inhibition of HIF-1α partially restored nuclear p53 localization, supporting the involvement of HIF-1α in this response. However, genetic approaches, such as *HIF1A* knockdown are required to establish a causal relationship and exclude potential off-target effects. Previous studies have reported direct interactions between HIF-1α and p53 under hypoxic stress in a context- and state-dependent manner [27]. However, because the Co-IP experiment was performed using whole-cell lysates, the present data do not confirm whether this association occurs in the cytoplasm. While stabilized HIF-1α may contribute to altered p53 localization, the present data also leave open the possibility that cytoplasmic p53 could contribute to the retention of WPSC-stabilized HIF-1α. Whether the association reflects direct binding or an indirect association remains to be determined. The different responses observed among the p53 variants may reflect variant-specific structural or regulatory properties, including protein conformation, stability, post-translational modifications, interacting partners, or nuclear transport. However, given the limited number of variants examined, the present data do not establish a general structure–function relationship [16,28,29]. The downstream effects of the WPSC-induced reduction in nuclear p53 were not examined in the present study and would require further investigations.

The observed association between HIF-1α stabilization and altered p53 localization may represent an additional route by which smoke-associated signaling affects tumor-suppressive responses, extending previous observations of HIF-1α–p53 crosstalk to WPSC exposure under normoxic conditions. These findings raise the possibility that HIF-1α inhibition may influence p53 function in the context of WPSC exposure, although this hypothesis requires further validation in chronic exposure and in vivo models.

The effects of WPSC on HIF-1α and p53 were most pronounced early and diminished by 24 h, indicating that the response to a single exposure is transient. This complements our earlier finding that prolonged WPSC exposure produces sustained signaling alterations [12] and suggests that acute and chronic exposures regulate stress responses differently.

## 4. Materials and Methods

### 4.1. Cell Culture

A549, H226, and H1299 cells were gifts from Dr. Fathia Mami Chouaib (Gustave Roussy, Villejuif, France), whereas H460 cells were purchased (AddexBio, San Diego, CA, USA; cat. no. C0016003; RRID: CVCL_0459). A549, H226, and H460 were maintained in RPMI-1640 medium (cat. no. 61870010; Gibco; ThermoFisher Scientific, Waltham, MA, USA), and H1299 cells were grown in DMEM (cat. no. 11965092; Gibco; ThermoFisher Scientific, Inc.) media. Both media were supplemented with 10% heat-inactivated fetal bovine serum (cat. no. 10270-106; Gibco; ThermoFisher Scientific, Inc.), 1% penicillin–streptomycin (cat. no. 15140-122; Gibco; ThermoFisher Scientific, Inc.), and 1% sodium pyruvate (cat. no. 11360-039; Gibco; ThermoFisher Scientific, Inc.). All cells were maintained at 37 °C in a humidified incubator containing 5% CO_2_ in normoxia (21% O_2_) or hypoxia (1% O_2_) using Whitley Hypoxystation (Don Whitley Scientific Ltd., Shipley, UK). Cells were routinely tested for mycoplasma contamination. PX-478 (cat. no. HY-10231; MedChemExpress, Monmouth Junction, NJ, USA) was used at 50 µM concentration alone or with WPSC for 4 h or 24 h in normoxia or in hypoxia.

### 4.2. Waterpipe Smoke Sampling and Analysis

Waterpipe smoke condensate was generated following an in-lab standardized procedure [12,30]. Briefly, 17.5 g of double-apple flavored tobacco (mou’assal) was placed in the waterpipe head and tightly covered with perforated aluminum foil. Two quick-light charcoal briquettes were positioned on top to heat the tobacco. Smoke was drawn through a robotic smoking apparatus (Sampler Tech LLC, Savage, MN, USA) programmed to mimic human puffing behavior, with a puff duration of 5 s and a 15 s interval between puffs, for a total of 120 puffs per session. During each puff, the smoke first passed through the water in the waterpipe base, as in conventional waterpipe use, before being collected on pre-conditioned glass wool fibers packed inside a T-shaped collection tube. All subsequent cell culture experiments were conducted using these prepared WPSC samples. Prepared condensates were aliquoted and stored at −80 °C until use. The stock WPSC was defined as 100%, from which working concentration were prepared by dilution in complete culture media. Cells were treated with 0.1% WPSC for 4 h or 24 h. The 0.1% concentration was selected based on dose–response MTT experiments showing no reduction in MTT signal at this concentration over 24–72 h. For combination experiments, cells were pretreated or co-treated with WPSC and additional agents as specified in the figure legend.

### 4.3. Hypoxia Detection

Cells were incubated with Image-iT™ Hypoxia Reagent Green (cat. no. I14833; ThermoFisher Scientific, Inc.) according to the manufacturer’s instructions. Briefly, cells (2.5 × 10^4^) were seeded onto glass-bottom 24-well imaging plates (cat. no. 0030741021; Eppendorf SE, Hamburg, Germany) treated with 0.1% WPSC or exposed to hypoxia (1% O_2_) for the indicated times and stained with the Image-iT™ Hypoxia Reagent Green (1 μM) for 30 min at 37 °C. Fluorescence images were captured using a LSM 800 with Airyscan confocal microscope (Carl Zeiss Microscopy GmbH, Jena, Germany). The percentage of green-positive cells was quantified (>200 cells) from at least three random fields per condition using ImageJ software version 1.54g (National Institutes of Health, Bethesda, MD, USA).

### 4.4. X-Ray Irradiation

To induce nuclear accumulation of p53, cells were irradiated with 4 Gy using an X-RAD 320 (Precision X-Ray, Inc., Madison, CT, USA) or pre-treated with WPSC for 4 h, then irradiated. The 4 Gy dose was selected as a moderate irradiation dose previously shown to activate p53-related responses in NSCLC cells [13]. After irradiation, cells were incubated under normoxic conditions for 4 h, 5 h or 24 h prior to downstream analysis.

### 4.5. Immunoblotting

Cells cultured in T75 flasks or 10 cm plates (confluency 70–80%) were washed once with ice-cold 1× PBS and lysed in 100 µL of RIPA buffer (150 mM NaCl, 0.1% Triton X-100, 0.5% sodium deoxycholate, 0.1% SDS, 50 mM Tris-Cl, pH 8.0) supplemented with a protease inhibitor cocktail (cat. no. P2714; Sigma-Aldrich; Merck KGaA, Darmstadt, Germany). Lysates were briefly sonicated on ice to shear DNA and reduce viscosity, and protein concentrations were determined using the Pierce BCA Protein Assay Kit (cat. no. 23225; ThermoFisher Scientific, Inc.). Equal amounts of protein (20–50 µg) were separated on 10–12% SDS-PAGE gels and transferred onto nitrocellulose membranes (cat. no. GE10600004; Sigma-Aldrich; Merck KGaA) over 4 h. Membranes were blocked in 5% BSA in TBST (10 mM Tris, pH 8.0, 150 mM NaCl, 0.1% Tween-20) for 1 h at room temperature, washed once with TBST, and incubated overnight at 4 °C with primary antibodies, followed by washing with TBST three times, then secondary antibodies for 1 h, and washed three times with TBST. All blots were imaged using the iBright FL1000 imaging system (Thermo Fisher Scientific, Waltham, MA, USA). Band intensity was quantified using ImageJ software version 1.54g (National Institutes of Health, Bethesda, MD, USA) and the results were normalized to the corresponding control. Primary antibodies are listed in Table S1.

### 4.6. Nuclear and Cytoplasmic Protein Fractionation

Cells (1 × 10^6^) were seeded in 10-cm dishes overnight and treated with 0.1% WPSC for 4 h or 400 µM H_2_O_2_ for 60 min, followed by replacement with fresh medium. H_2_O_2_ (400 µM, 60 min) was used as an acute oxidative-stress condition, based on previously reported short-term H_2_O_2_ treatments in A549 cells [31,32]. Cells were collected by trypsinization, washed with ice-cold PBS, and resuspended in 350 µL cytoplasmic extraction buffer (10 mM HEPES, pH 7.9, 10 mM KCl, 1.5 mM MgCl_2_, 1 mM EDTA, 0.5 mM DTT, 0.05% NP-40, and protease inhibitors). Following incubation on ice for 5–8 min, samples were centrifuged at 1000× *g* for 10 min at 4 °C, and the supernatant was collected as the cytoplasmic fraction. The nuclear pellet was washed once with cytoplasmic buffer lacking NP-40 and resuspended in 70 µL nuclear extraction buffer (20 mM HEPES, pH 7.9, 420 mM NaCl, 1.5 mM MgCl_2_, 0.2 mM EDTA, 0.5 mM DTT, and protease inhibitors). Nuclear proteins were extracted for 30 min with vortexing every 5 min and sonication every 10 min, followed by centrifugation at 14,000× *g* for 30 min at 4 °C. The supernatant was collected as the nuclear fraction. For fraction comparisons, equal sample volumes were loaded from both the cytoplasmic and nuclear lysates (prepared at a 5:1 cytoplasmic-to-nuclear extraction ratio). Fraction purity was assessed by Western blotting using histone H3 and α-tubulin as nuclear and cytoplasmic markers, respectively.

### 4.7. Immunofluorescence

Cells (5.0 × 10^4^) grown on glass coverslips (12 mm) were first fixed with 4% paraformaldehyde (cat. no. 28906; ThermoFisher Scientific, Inc.) in 1× PBS for 15 min at room temperature. Following fixation, cells were rinsed with PBS and permeabilized using 0.1% Triton X-100 in PBS for 15 min. Non-specific binding sites were blocked by incubating the cells in 2% BSA in PBS for 1 h at room temperature. Cells were then incubated with primary antibodies for 1 h at room temperature as specified in each figure, followed by three 5 min washes in PBS, and subsequently incubated with the appropriate secondary antibodies for 1 h at room temperature in the dark, followed by three 5 min washes in PBS. Where indicated, F-actin was stained using Alexa Fluor 488-conjugated Phalloidin (cat. no. A12379; Thermo Fisher Scientific, Inc.), and nuclei were counterstained with DAPI. Finally, coverslips were mounted on glass slides using ProLong Gold Antifade Reagent (cat. no. P36930; ThermoFisher Scientific, Inc.). Coverslips were imaged using a Zeiss LSM 800 confocal microscope equipped with Airyscan (Carl Zeiss Microscopy GmbH, Jena, Germany) for high-resolution visualization. Antibodies are listed in Table S1.

### 4.8. RNA Extraction/cDNA and RT-qPCR

Total RNA was extracted using Easy-BLUE™ Total RNA Extraction Solution (cat. no. 17061; iNtRON Biotechnology, Seongnam, Republic of Korea.) according to the manufacturer’s instructions. RNA concentration and purity were assessed using a NanoDrop spectrophotometer (Thermo Fisher Scientific, Waltham, MA, USA). cDNA was synthesized from 1 µg of total RNA using the High-Capacity cDNA Reverse Transcription Kit with RNase Inhibitor (cat. no. 4374966; Applied Biosystems; Thermo Fisher Scientific, Inc.) in a final reaction volume of 20 µL. Reverse transcription was performed at 25 °C for 10 min, 37 °C for 120 min and 85 °C for 5 min, followed by a hold at 4 °C.

RT-qPCR was performed using SYBR™ Green PCR Master Mix (2X; cat. no. 4309155; Applied Biosystems; Thermo Fisher Scientific, Inc.) on a CFX384 Touch Real-Time PCR Detection System (Bio-Rad Laboratories, Inc., Hercules, CA, USA). Reactions were performed in a final volume of 10 µL, containing 5 µL of 2X SYBR Green PCR Master Mix, 400 nM of each forward and reverse primer, 1 µL of cDNA, and nuclease-free water. The thermal cycling conditions consisted of 50 °C for 2 min and 95 °C for 10 min, followed by 40 cycles of 95 °C for 15 s and 60 °C for 1 min. Relative gene expression was calculated using the 2^−ΔΔCq^ method as described by Livak and Schmittgen [33], with β-actin (*ACTB*) used as the reference gene. Primer sequences are provided in Table S2.

### 4.9. Plasmid Transfection

H1299 cells were transiently transfected with plasmids encoding wild-type p53 (Addgene plasmid #16434) or the mutant variants R248W, R273H and R175H (Addgene plasmids #16437, #16439 and #16436, respectively). Transfections were performed using jetPEI^®^ transfection reagent (Polyplus-transfection, Illkirch, France) according to the manufacturer’s instructions. For each condition, 10 µg of plasmid DNA was complexed with jetPEI^®^ at a 2:1 jetPEI-to-DNA ratio and added to cells in complete growth medium. Cells were incubated for 24 h following transfection, after which the medium was replaced with fresh complete medium. Cells were subsequently treated with 0.1% WPSC for 4 h before downstream analysis.

### 4.10. Co-Immunoprecipitation (Co-IP)

Following 4 h WPSC exposure, whole-cell lysates from non-treated and WPSC-treated cells were prepared for co-immunoprecipitation analysis. Protein–protein interactions were assessed using the Pierce Classic IP Kit according to the manufacturer’s protocol (cat. no. 26146; ThermoFisher Scientific, Inc.). Briefly, cells were lysed in immunoprecipitation buffer supplemented with protease inhibitor cocktail (cat. no. P2714-1BTL; Sigma-Aldrich). Equal amounts of protein lysate (1 mg) were incubated with 2 µg anti-p53 7F5 antibody overnight at 4 °C to allow for immune complex formation. The following day, Protein A/G agarose beads were added and incubated for 1 h to capture antibody-bound complexes. Beads were subsequently washed three times with IP buffer to remove non-specific interactions. Bound proteins were then eluted in Laemmli sample buffer, resolved by SDS-PAGE, and analyzed by immunoblotting for p53 and HIF-1α.

### 4.11. Statistical Analysis

All experiments were performed in at least three independent biological replicates. Data are presented as the mean ± standard deviation (SD). Statistical analyses were performed using GraphPad Prism 9. Depending on the experimental design, statistical significance was assessed using one-way or two-way analysis of variance (ANOVA), followed by the appropriate multiple-comparisons procedure as specified in the corresponding figure legends. *p* ≤ 0.05 or q ≤ 0.05 for analyses using false discovery rate correction was considered statistically significant.

## 5. Conclusions

WPSC induces normoxic accumulation of HIF-1α and is associated with transient impairment of p53 nuclear localization in NSCLC cells, with attenuation of irradiation-induced p53 target gene expression demonstrated in A549 cells. Pharmacological inhibition of HIF-1α partially reverses the change in p53 localization, while co-immunoprecipitation supports an association between HIF-1α and p53 following WPSC exposure. These findings support the involvement of HIF-1α in WPSC-associated alterations in p53 signaling, although the molecular basis and biological consequences require further investigation.

## Data Availability

The original contributions presented in this study are included in the article/Supplementary Material. Further inquiries can be directed to the corresponding author.

## Supplementary Materials

The following supporting information can be downloaded at: https://www.mdpi.com/article/10.3390/ijms27198927/s1.
