# Supplementary material for: Waterpipe Smoke Condensate Impairs p53 Nuclear Localization in Association with HIF-1α Stabilization
Source: Int J Mol Sci. 2026 Oct 8;27(19):8927. doi: 10.3390/ijms27198927 (PMC13665274; doi:10.3390/ijms27198927)
Supplement: Supplementary file 1 [file ijms-27-08927-s001.zip › Supplementary Figures.pdf]

Figure S1:

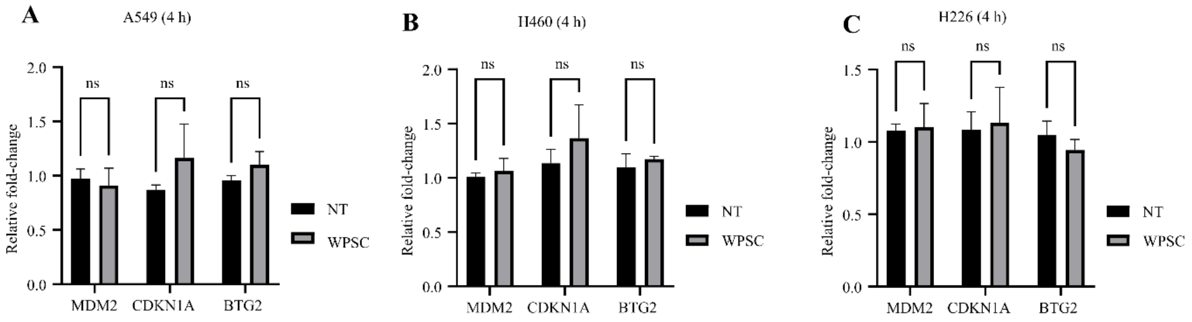

Figure S1. Effect of WPSC exposure on p53 transcriptional activity in NSCLC cells. (A) A549, **(B)** H460 and **(C)** H226 cells were treated with 0.1% WPSC for 4 h under normoxic conditions. The mRNA expression levels of the p53 target genes *MDM2*, *CDKN1A* and *BTG2* were assessed by RT-qPCR and normalized to β-actin (*ACTB*). Data are presented as the mean ± SD from at least three independent experiments. Statistical analysis was performed using two-way ANOVA. ns, not significant.

Figure S2:

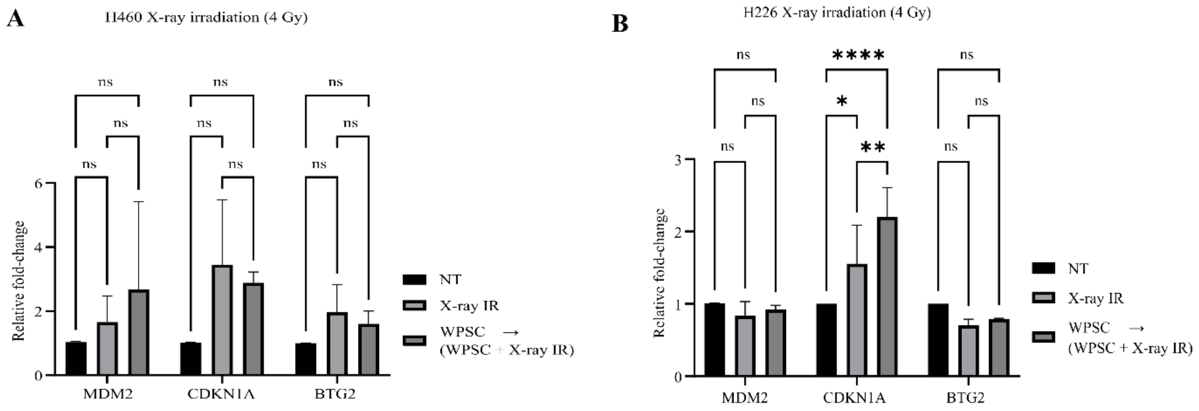

Figure S2. Differential induction of p53 target genes following X-ray irradiation in H460 and H226 cells. (A) H460 and **(B)** H226 cells were exposed to X-ray irradiation (4 Gy) alone or following 4 h WPSC pretreatment and were harvested 5 h after irradiation. The mRNA expression levels of the p53 target genes *MDM2*, *CDKN1A* and *BTG2* were assessed by RT-qPCR and normalized to β-actin (*ACTB*). Data are presented as the mean ± SD from at least three independent experiments. Statistical analysis was performed using two-way ANOVA followed by the Benjamini-Hochberg false discovery rate (FDR) procedure for multiple comparisons, comparing treatment conditions within each gene. Statistical significance was determined at an FDR of 5% ($q \leq 0.05$). **$q \leq 0.01$ and ****$q \leq 0.0001$; ns, not significant.

Figure S3:

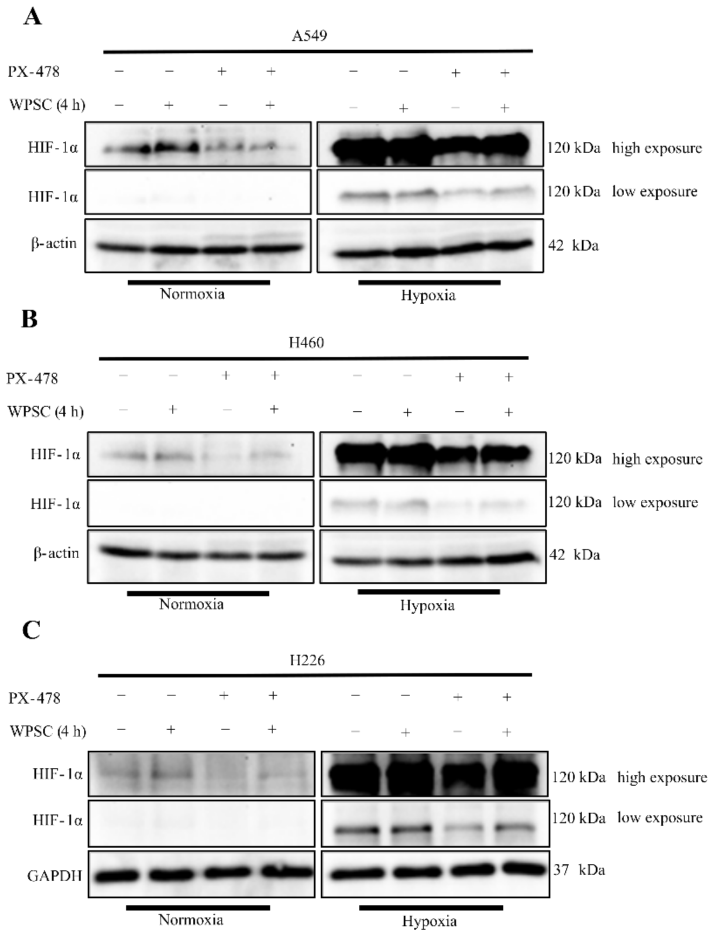

Figure S3. PX-478 reduces HIF-1α protein accumulation in WPSC-treated NSCLC cells. Representative western blot analysis of HIF-1α protein expression in **(A)** A549, **(B)** H460 and **(C)** H226 cells treated with 0.1% WPSC, 50 μM PX-478 or their combination for 4 h under normoxic or hypoxic conditions. High- and low-exposure images of the same HIF-1α immunoblots are shown to allow visualization of differences in protein abundance. β-actin was used as the loading control for A549 and H460 cells, whereas GAPDH was used for H226 cells.
