# Supplementary material for: Waterpipe Smoke Condensate Impairs p53 Nuclear Localization in Association with HIF-1α Stabilization
Source: Int J Mol Sci. 2026 Oct 8;27(19):8927. doi: 10.3390/ijms27198927 (PMC13665274; doi:10.3390/ijms27198927)
Supplement: Supplementary file 1 [file ijms-27-08927-s001.zip › Supplementary tables.pdf]

Table S1. Probes and primary and secondary antibodies used in the present study.

Reagent	Catalog no.	Company	Dilution/amount	Application
Rabbit anti-HIF-1α (D2U3T)	14179	Cell Signaling Technology	1:1,000	WB
Rabbit anti-GAPDH (14C10) mAb	2118	Cell Signaling Technology	1:1,000	WB
Mouse anti-β-actin (C4)	sc-47778	Santa Cruz Biotechnology	1:2,000	WB
Rabbit anti-α-tubulin antibody	2144	Cell Signaling Technology	1:1,000	WB
Mouse anti-HIF-1α (D5F3M)	79233	Cell Signaling Technology	1:400	IF
Alexa Fluor 488 phalloidin (F-actin)	A12379	Thermo Fisher Scientific	1:1,000	IF
Mouse anti-p53 (DO-7) monoclonal antibody	48818	Cell Signaling Technology	1:400 (IF); 1:1,000 (WB)	IF; WB
Rabbit anti-histone H3	9715	Cell Signaling Technology	1:2,000	WB
Rabbit anti-p53 (7F5) mAb	2527	Cell Signaling Technology	1:1,000 (WB); 2 µg (IP)	WB; IP
HRP-conjugated mouse anti-p53 (DO-1) antibody	ab204452	Abcam	1:2,000	WB
Goat anti-rabbit Alexa Fluor 488	A11034	Thermo Fisher Scientific	1:2,000	IF
Goat anti-rabbit Alexa Fluor 568	A-11011	Thermo Fisher Scientific	1:1,000	IF
Goat anti-mouse Alexa Fluor 568	A11004	Thermo Fisher Scientific	1:1,000	IF
Anti-mouse IgG HRP-linked antibody	7076S	Cell Signaling Technology	1:10,000	WB
Anti-rabbit IgG HRP-linked antibody	7074	Cell Signaling Technology	1:10,000	WB

WB, western blotting; IF, immunofluorescence; IP, immunoprecipitation; HRP, horseradish peroxidase; mAb, monoclonal antibody.

Table S2. Primer sequences used for RT-qPCR.

Gene	Primer	Sequence (5′→3′)
ACTB	Forward	TCCTTCCTGGGCATGGAGT
ACTB	Reverse	AGCACTGTGTTGGCGTACAG
PGK1	Forward	TCTCATGGATGAGGTGGTGA
PGK1	Reverse	CTTCCAGGAGCTCCAAACTG
VEGFA	Forward	GGGCAGAATCATCACGAAGT
VEGFA	Reverse	TGGTGATGTTGGACTCCTCA
SLC2A1	Forward	GGCCAAGAGTGTGCTAAAGA
SLC2A1	Reverse	CGACTCTCTTCCTTCATCTCCT
MDM2	Forward	ACCACCTCACAGATTCCAGCTT
MDM2	Reverse	GCACCAACAGACTTTAATAACTTCAA
CDKN1A	Forward	GTCACTGTCTTGTACCCTTGTG
CDKN1A	Reverse	GGCGTTTGGAGTGGTAGAAA
BTG2	Forward	CCTGTGGGTGGACCCCTAT
BTG2	Reverse	GGCCTCCTCGTACAAGACG
